# Pleomorphic rhabdomyosarcoma infiltrating thoracic spine in a 59-year-old female patient: Case report

**DOI:** 10.3205/iprs000113

**Published:** 2017-07-24

**Authors:** Matthias Spalteholz, Jens Gulow

**Affiliations:** 1 Department of Spine Surgery, Helios Park-Klinikum, Leipzig, Germany

**Keywords:** pleomorphic rhabdomyosarcoma, soft tissue sarcoma, thoracic spine, en-bloc spondylectomy

## Abstract

Rhabdomyosarcoma (RMS) represents a malignant tumor of skeletal muscle cells arising from rhabdomyoblasts. RMS represents the most common soft tissue sarcoma in children. In adults it is uncommon and accounts for less than 1% of all malignant solid tumors. While treatment protocols are well known for children, there is no standardized regimen in adults. This is one reason, why the outcome in adults is worse than in children. We present the case of a 59-year-old female patient with pleomorphic rhabdomyosarcoma (PRMS) infiltrating the thoracic spine. Multimodality treatment was performed including en-bloc resection, adjuvant multidrug chemotherapy and radiation beam therapy. The patient was tumor free and had no relapse within 6 month follow-up.

## Zusammenfassung

Das Rhabdomyosarkom (RMS) stellt einen bösartigen Tumor der Skelettmuskulatur dar, der aus Rhabdomyoblasten entsteht. Das RMS stellt das häufigste Weichteilsarkom bei Kindern dar, bei Erwachsenen ist es selten und macht weniger als 1% aller bösartigen soliden Tumoren aus. Während Behandlungsprotokolle für Kinder etabliert sind, gibt es kein standardisiertes Regime bei Erwachsenen. Dies ist ein Grund, warum das Outcome bei Erwachsenen schlechter ist als bei Kindern. Wir stellen den Fall einer 59-jährigen Patientin mit einem pleomorphen Rhabdomyosarkom (PRMS) vor, welches die Brustwirbelsäule infiltriert. Es wurde ein multimodales Behandlungskonzept einschließlich En-Bloc-Resektion, adjuvanter Chemotherapie und Strahltherapie durchgeführt. Die Patientin war postoperativ tumorfrei und hatte kein Rezidiv innerhalb der ersten 6 Monate.

## Introduction

Sarcoma is a rare cancer with a reported incidence of 1% of all malignancies and 20% of all mesenchymal malignancies. 80% arise from soft tissues and 20% from bone [1], [2]. Sarcomas may be related to genetic syndromes [3], e.g.: Li-Fraumeni syndrome [4], neurofibromatosis [5], Beckwith-Wiedemann syndrome [6], Costello syndrome [7]. RMS is the most common soft tissue sarcoma in children, 80% occur in children younger than 12 years [8], [9], [10], [11]. RMS is uncommon in adults. The prevalence is 2–5% of all sarcomas in adults older than 45 [12], [13]. PRMS is a histological subtype, which was first reported as classic RMS in 1946 [14]. PRMS arises predominantly in the extremities of adult males with mean age of 49. The tumor is mostly asymptomatic or presents with signs and symptoms that are associated with tumor site [15]. 20% of patients present with distant metastasis: mostly in lung 39%, bone marrow 32%, lymph nodes 30%, bones 27% [16]. Due to advanced disease presentation and the absence of standardized treatment protocols the outcome is worse in adults [17], [18]. The 5-year overall survival rate is 27% in adults [19]. Its prognosis is significantly worse than other pleomorphic sarcomas [20]. 

## Case report

**Figure 1:**
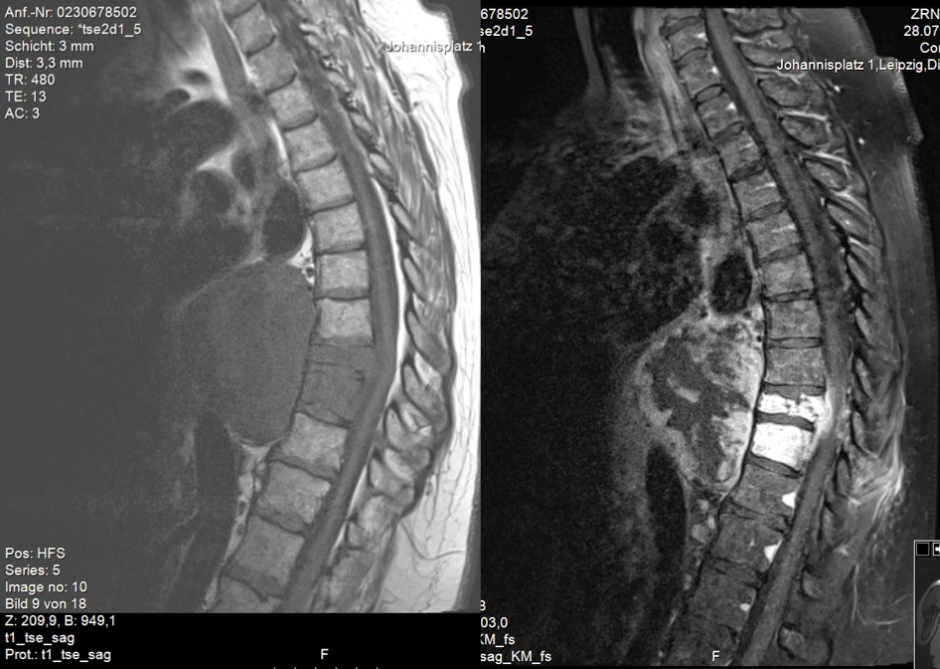
Sagittal MR image of the thoracic spine. Left: T1-weighted image. The tumor represents as a hypointense mass infiltrating the spine with spinal canal penetration. Right: T1-weighted, contrast enhanced image. Inhomogeneous gadolinium distribution.

**Figure 2:**
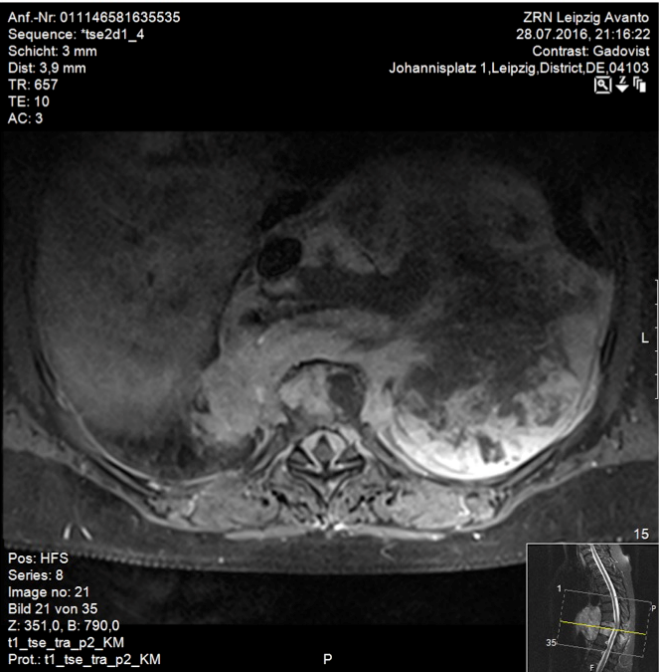
T1-weighted, contrast enhanced transverse MR image of the thoracic spine. Inhomogeneous gadolinium distribution. Solid mass in the posterior inferior mediastinum with spinal canal infiltration and myelon compression.

**Figure 3:**
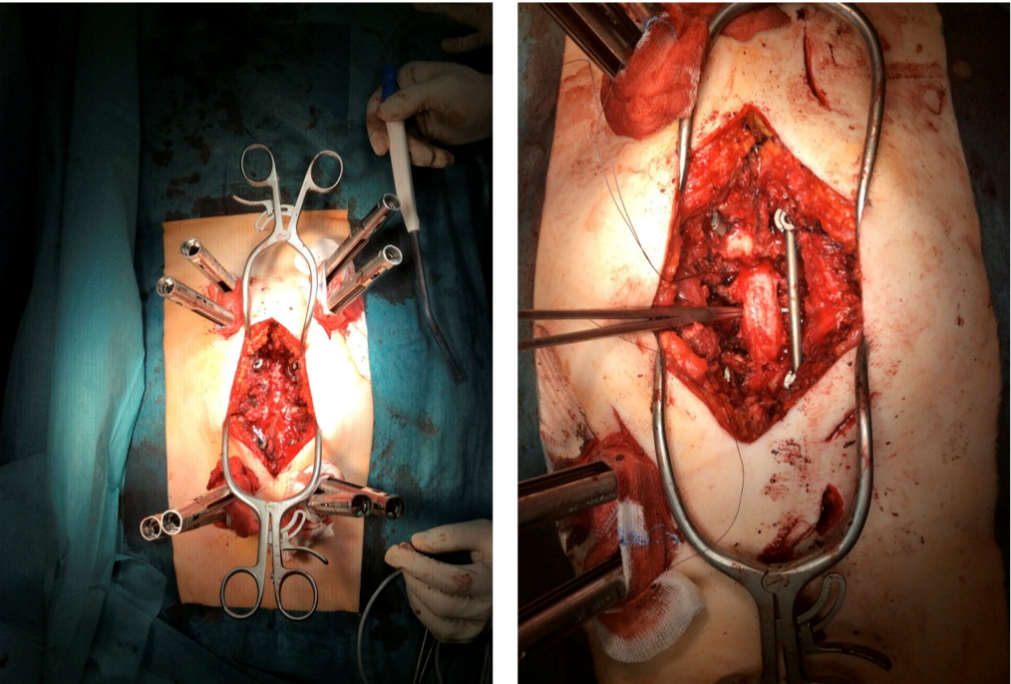
Posterior long segment stabilization. Left: To decrease length of incision and muscle trauma, the cranial and caudal screws were placed in a percutaneous fashion. Right: Complete resection of the posterior bony elements of Th9 and 10, with tumor debulking and spinal canal clearance. The spinal nerves T9 and T10 were cut bilaterally.

**Figure 4:**
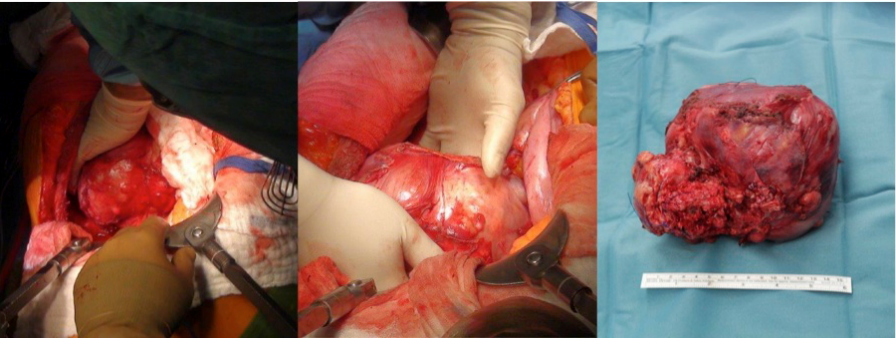
Anterior approach: Thoracolumbophrenotomy with en-bloc resection

**Figure 5:**
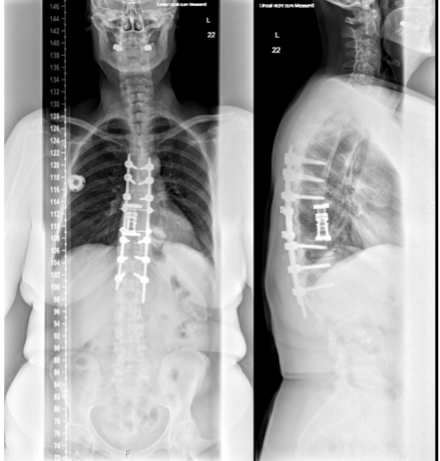
Postoperative X-ray (a.p. and lateral view). Posterior long segment pedicle screw stabilization and anterior reconstruction via expandable cage.

**Figure 6:**
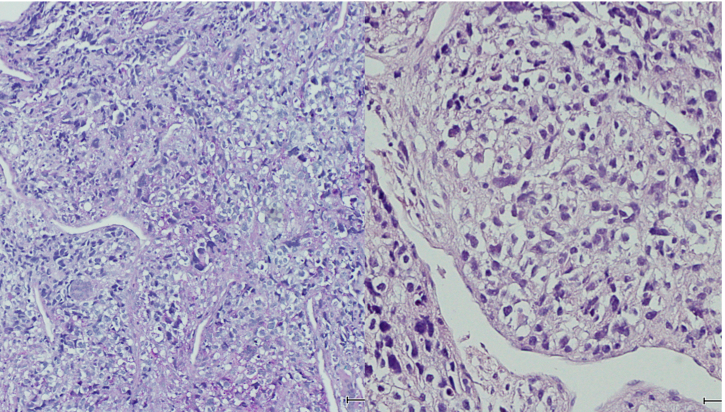
Mononuclear tumor cells with a mix of giant cells, spindle cells and round shaped cells. Hyperchromatic nuclei, partly pale and deeply eosinophilic cytoplasm.

 A 59-year-old woman presented with persistent pain in the thoracic spine after low energy trauma 6 weeks ago. X-ray imaging showed vertebra compression fracture of Th9 with kyphosis of 20°. The patient had a history of three strokes, 15 and 13 years ago, with residual paralysis on the right side. She did not report of new neurological disability after trauma: no new motor dysfunction, no long tract signs, neither bladder nor bowel dysfunction. There was no history of malignancy in her family. MRI studies revealed a solid mass of 16x14x12 cm in the inferior posterior mediastinum on the left side with infiltration of Th9 and Th10 vertebral body and spinal canal infiltration with myelon compression (Figure 1 (Fig. 1), Figure 2 (Fig. 2)). Staging procedure including total spine MRI, abdominal and chest computed tomography (CT), cranial CT, and PET CT did not show distant metastasis. The interdisciplinary tumor board decided to perform a transthoracic needle aspiration biopsy first. The histopathological examination could not define a clear diagnosis. Therefore an open biopsy was performed. This result was not clear, too. At last, pleomorphic rhabdomyosarcoma or liposarcoma with rhabdomyogenic dedifferentiation were discussed. The proliferation index Ki67 was 70%, meaning high-grade sarcoma. Trimodality treatment was proposed: surgery with “en-bloc” resection followed by adjuvant multidrug chemotherapy and radiation beam therapy. The day after biopsy, the patient suddenly developed an ascending sensory spinal cord injury. We decided for a two-stage surgery, starting with immediate posterior decompression and stabilization: We performed a posterior decompression with complete resection of the posterior parts of the Th9 and Th10 vertebra with spinal canal clearance. Long segment stabilization was performed three levels above and three levels below (Figure 3 (Fig. 3)). After a few days of recovery the anterior procedure followed (Figure 4 (Fig. 4)). Via thoracolumbophrenotomy an “en bloc” resection including TH9 and Th10 vertebral body as well as 2 level cage reconstruction of the anterior column was performed (Figure 5 (Fig. 5), Figure 6 (Fig. 6)). The patient recovered quite well after surgery: no problems of wound healing, no new neurological deficits, sensory deficit improved. Adjuvant chemotherapy started 4 weeks after surgery with the first of 3 cycles in a 21 days period. Unfortunately chemotherapy had to be reduced and later stopped due to major side effects. A radiation beam therapy with total radiation dose of 60 Gy followed. The patient did well at 3 month and 6 month follow-up, showing no new neurological deficits, less pain. MR imaging after 6 month did not show local recurrence and PET CT did show no proof of relapse. 

## Discussion

RMS accounts for less than 1% of all malignant solid tumors in adults [12], [13], [21], [22], [23], [24]. RMS can arise anywhere, but the most common area is head and neck (40%), genitourinary tract (25%), extremities (20%), chest wall and retroperitoneum [21]. Four subtypes are distinguished: embryonal, alveolar, pleomorphic and mixed type [21]. The embryonal type (ERMS) is the most common one (>70%) with characteristic round cells looking like lymphocytes and spindled cells with elongated nuclei and eosinophilic cytoplasm. The alveolar type (ARMS, 10–20%) involves areas of spaces lined by non cohesive round or oval cells [25]. Pleomorphic RMS (PRMS) is the most common subtype in adults, which shows large pleomorphic rhabdomyoblasts with eosinophilic cytoplasm. Three morphologic variants are described: classic, round cell, spindle cell [14], [24], [26], [27]. The mixed type involves more than one histologic subtype. The histopathological diagnosis is challenging, therefore an experienced pathologist is indispensable. PRMS has at least one specific skeletal muscle marker (myoglobin, nuclear MyoD1, nuclear myf4, fast myosin) and non-specific markers (desmin, myogenin). Myoglobin (95%) and fast skeletal muscle myosin (80%) are the most sensitive marker for PRMS [24]. As the classification of sarcomas, especially subtyping, is difficult, molecular testing methods become important. 90% of alveolar RMS show characteristic translocation involving one of the PAX genes and the forkhead FKHR gene. ERMS shows an overexpression of PAX 7 gene. 70–80% of ARMS demonstrate an expression of PAX 3-FKHR or PAX7-FKHR translocation [28]. PAX-FKHR is a pleiotropic fusion protein that stimulates proliferation, induces angiogenesis, inhibits apoptosis and inhibits terminal differentiation [29]. In our case, the histological and molecular examination was partly contradictory, even after referencing. The tumor cells showed desmin, myogenin and MyoD1 positive reaction in immunohistochemistry, indicating rhabdomyosarcoma. There was no lipogenic component at all. On the other hand molecular pathological testing revealed FKHR-Gen expression of only 2%, excluding chromosomal translocation at 13q14.11. This fact speaks against up RMS and for liposorcoma. The MDM2 cluster amplification using fluorescence in situ hybridization (FISH), made a liposarcoma with rhabdomyogenic dedifferentiation possible. MR imaging is the method of choice to describe localization and the extent of such lesions. In relation to muscle structures, RMS represents as iso- to hypointensity in T1-weighted images and hyperintensitiy in T2-weighted images. Gadolinium enhancement is seen with homogenous as well as inhomogeneous distribution [30]. To evaluate metastatic spinal lesions, total spine MRI is recommended [31]. The European cooperative group (COG), formerly Intergroup Rhabdomyosarcoma Study Group (IRSG), categorized patients into 4 risk groups dependent on a clinical staging system: Low-risk, standard-risk, high-risk, and very-high risk. Survival rates of 83% with stage I, 70% stage II, 52% stage III and 20% with stage IV disease are reported [18], [32], [33], [34]. The absence of distant metastases, favorable anatomic sites, complete surgical removement and tumor size <5 cm are beneficial prognostic factors [22], [34], [35], [36]. As mentioned above, the outcome of RMS in adults is worse than in children due to less cases and missing of standardized protocols. Most published studies for adults are retrospective case series with limited data. Treatment protocols of ARMS and ERMS in pediatrics may be used in adults, but there is no evidence [12], [22], [37], [38], [39]. The therapeutical regimen is a multidisciplinary concept with focus on local (surgery, radiation) and systemic tumor control (chemotherapy). The multimodality concept is superior to any single modality therapy [32], [40]. The role of surgery is dependent on tumor site and tumor size. Surgery alone is never curative, but a superior outcome has been suggested when initial complete or marginal resection or even debulking is performed. Functionality and cosmetic sparing surgery is important [41], [42], [43], [44]. “En bloc” spondylectomy provides good oncological outcome with lower incidence of local recurrence and is recommended for solitary spinal lesions as well [45], [46], [47]. In proper sense, wide excision (en bloc) is technically infeasible, due to the anatomical conditions (relationship of visceral structures and great vessels to the spine). In general, all children are treated with chemotherapy (duration 6–12 month in a pulse (2–5 days) or cyclic fashion (3–4 weeks). Most have surgery and radiation therapy [48], [49], [50]. The most used chemotherapy combination in children is a three-drug therapy with vincristine, actinomycin-D, and cyclophosphamide (VAC). Another combination includes vincristine, doxorubicin, and cyclophosphamide. The response to chemotherapy is as high as 85% [13]. The main difference between children and adults is the use of ifosfamide instead of cyclophosphamide and use of doxorubicin instead of actinomycin-D. Our patient received a multidrug combination of 8x ifosfamide (1125 mg/m² day 1–5), epirubicin (45 mg/m² day 2+3) and pegfilgastrim (6 mg at day 6). Due to major side effects (severe pancytopenia, ifosfamide-induced myoclonia) the doses had to be reduced (cycles 2 and 3: 5x ifosfamide 1500 mg/m² day 1–5, epirubicin 37.5 mg/m² day 2+3 and pegfilgastrim 6 mg at day 6). After the third cycle it had to be stopped. Radiation therapy with peak doses of 40–50 Gy is recommended in children at incomplete resection or lymph node involvement in ERMS and in any case of ARMS, even if it is resected completely. In our case an intensity-modulated photon radiation therapy (IMRT) was implemented with a total radiation dose of 60 Gy in 25 days (two times 1.2 Gy/day with total of 50 irradiations). The reported loco-regional treatment failure is up to 50% and the overall 3 year failure – free survival rate for all patients is 77% [51], [52], [53], [54], [55]. 50% of all patients will die within one year and 90% within 5 years of relapse [56]. Therefore MR imaging should be repeat every 6 month for the first 2 years for high grade or large lesions [57].

## Conclusion

Pleomorphic rhabdomyosarcoma is a rare disease. There are only a few cases with spinal involvement in adults. Like in our case, the histopathological classification is often difficult, therefore referencing by specialized pathologists and molecular pathological methods are indispensable. An individual case discussion in a specialized multidisciplinary tumor board as well as a multimodality treatment concept is crucial to improve survival rates. Frequent follow-up examinations are necessary due to high rates of treatment failure.

## Notes

### Competing interests

The authors declare that they have no competing interests.
